# Comparison of highly purified urinary versus recombinant FSH: Effect on ART outcomes in polycystic ovary syndrome

**Published:** 2012-05

**Authors:** Farnaz Sohrabvand, Shahrzad Sheikhhassani, Maryam Bagheri, Fedyeh Haghollahi, Maryam Shabihkhani, Mamak Shariat, Manijeh Nasr Esfahani

**Affiliations:** 1 *Department of Obstetrics- Gynecology and Infertility, Vali-e-Asr Hospital, Imam Khomeini Hospital Complex, Tehran University of Medical Sciences, Tehran, Iran.*; 2 *Vali-e-Asr Reproductive Health Research Center, Tehran University of Medical Sciences, Tehran, Iran.*; 3 *Maternal- Fetal and Neonatal Research Center, Tehran University of Medical Sciences, Tehran, Iran.*

**Keywords:** *ART outcome*, *PCOS*, *Highly purified urinary FSH*, *Recombinant FSH*, *Clinical pregnancy rate*

## Abstract

**Background**: Polycystic ovary syndrome (PCOS) is a common cause of ovulatory disorders and infertility with high LH to FSH ratio. In order to prevent further increase of LH and follicle atresia, different regimens for ovulation induction have been recommended using FSH alone.

**Objective:** This study was performed in PCOS patients to compare ART outcomes in cycles induced by FSH alone, using either recombinant or urinary products.

**Materials and Methods:** In a randomized trial, from 623 patients who underwent down regulation with GnRH analogue in a long protocol, 160 PCOS patients were randomly divided into two groups of 80. Group A received 150 IU/d recombinant FSH (Gonal-F) and group B 150 IU/d urinary FSH (Fostimon).

**Results:** 33 cases (41.2%) in group A and 36 (45%) in group B achieved clinical pregnancy, which was not significantly different (p=0.67). Total number of oocytes retrieved (13.03±5.56 vs. 14.17±4.89, p=0.17), quality and number of embryos (7.42±3.35 vs. 7.63±3.28, p=0.68) and OHSS rate were similar in group A compared to group B. Endometrial thickness which was 9.66±1.67 mm in group A and 10.36±1.35 mm in group B, showed a significant difference (p=0.004).

**Conclusion:** It seems that in PCOS patients, both pure FSH products used for controlled ovarian hyperstimulation have similar effects on ART outcome and can be used according to availability and patient acceptance without significant difference.

## Introduction

Ovulation disorders are the cause of infertility in 30-40% of cases (1), with polycystic ovary syndrome (PCOS) being the most frequent disorder. Increased serum LH levels with suppression of FSH function within the ovary contribute to one of its major endocrine characteristics (2, 3). 

This biochemical imbalance creates a challenge in ovulation induction in infertile patients presenting with this problem (4). Since many years both FSH and human menopausal gonadotropin (hMG) products have been successfully used for ovulation induction in ovulatory disorders including PCOS (5), but it is an area of debate that in conditions like PCOS when there is a high level of endogenous LH, FSH alone is a better choice. 

It is a common concept that further increase of LH may prevent follicular maturity and contrarily lead to atresia (6, 7). PCOS patients are also very sensitive to gonadotropin stimulation (8) with excessive follicular development, leading to ovarian hyperstimulation syndrome (OHSS) and multifetal pregnancy (9). 

Hence the induction of ovulation in these patients still presents a challenge and requires further research to elucidate better methods and compounds to be used with good results and less complications (10). The first commercially available gonadotropin, hMG (Pergonal®), which was purified from the urine of postmenopausal women and contained approximately equal amounts of FSH and LH activity, was introduced in 1960s. There after many new products have been developed for induction of ovulation in infertile patients (1). 

In 1986, highly purified FSH with approximately 4% impurities and less than 0.1% LH (uFSH-HP) became available for clinical use.In 1988 recombinant FSH (rFSH) was prepared by transfecting Chinese hamster ovary cell lines with both FSH subunit genes (11). Both rFSH and uFSH were supposed to be more suitable for ovarian hyperstimulation (COH) protocols in women with PCOS since these women have elevated levels of endogenous LH. However to date there is no convincing evidence to support that in these patients FSH alone is more effective than hMG. 

Nevertheless it has been shown that FSH alone protocols can be safer in patients with a past history of ovarian hyperstimulation syndrome (12), and therefore preferable in PCOS patients. Overall according to the existing data no significant advantage of either rFSH or uFSH-HP in terms of ART outcome has been shown (13). Since this issue has not been addressed in PCOS patients who are in fact one of the target groups for these products, this study was designed to compare the ART outcome between the two pure FSH preparations, Gonal-F (recombinant FSH) and Fostimon (urinary hp-FSH) available in Iran.

## Materials and methods

**Study design**

This was a prospective randomized controlled trial which was performed from October 2008-December 2009 at the Infertility Department of Vali-e-Asr Hospital as a gynecology resident thesis after being accepted by the Research Committee of the Tehran University of Medical Sciences and also obtaining ethical approval from the Faculty of Medicine Ethics Committee (Ref no:835). 

**Patient selection**

From among 623 patients undergoing ART cycles during the study period, 235 women were diagnosed with PCOS according to Rotterdam criteria (14) aged 20-35 years. After exclusion of PCOS patients with body mass index (BMI) >30 kg/m^2^ (n=10) and those with other infertility problems i.e endometriosis (n=9) and male factor (n=45) 160 patients with a BMI range of 18-30 were included if they had no underlying medical illnesses and no contraindications for pregnancy. 

Patients with other ovulation disorders such as hypo and hyper-gonadotropic hypo gonadism, hyper-prolactinemia, thyroid disorders, ovarian or adrenal neoplasms, Cushing syndrome and infertility due to causes other than PCOS and a previous history of inappropriate ovarian response to stimulation with gonadotropins (poor responders) were excluded.

**Randomization**

After obtaining written consent they were allocated by the clinic secretary to one of two groups by simple random sampling, using a random numbers table. The clinician, ultra sonographer, embryologist and statistician were all blinded. In order to detect a change of 8-10% in metaphase II oocytes which leads to a power of 80% a sample size of 80 in each study group was calculated. Data collection was done via questionnaires completed by clinic staff and laboratory analyses.

**Treatment Protocol**

Baseline FSH, LH and testosterone serum levels were measured for all patients in their previous cycles. All patients underwent pituitary down regulation receiving a once daily subcutaneous dose of 0.2cc Buserelin (Suprefact, Hoechst, AG-Germany), a short-acting GnRH analog from the 21^st^ day of their cycles with oral contraceptive pills (OCP) pretreatment. 

After stopping OCP and at least 12 days of pituitary suppression, the patients were randomly allocated to group A who received recombinant FSH (Gonal-F, Serono, Switzerland) or group B who were treated with highly purified urinary FSH (Fostimon, IBSA, Switzerland) each at a dose of 150 IU/d for the first six days when a vaginal sonoghraphic exam was performed and in case of appropriate response, the patients underwent sonography every other day until they had at least two follicles ≥18 mm and at least two other follicles with a diameter >16 mm when they received 10000 IU HCG. If their response was insufficient, on the seventh day they received 1-2 additional ampoules. 

The patients were also asked to report symptoms such as abdominal discomfort, nausea, vomiting, diarrhea and the presence of more than 20 follicles in the ovary were registered which were considered as signs of OHSS. In group A, 21 (26.25%) and in group B, 24 (30%) patients received Metformin. Oocyte pickup was performed 34 to 36 hours following HCG administration. Oocyte maturation was assessed with the criteria described by Veeck (15). After the ICSI procedure, embryos were scored according to the morphologic appearance of their blastomers and fragmentation (16). 

Embryo transfer was performed on day three of ovum pickup with no more than 3 embryos being transferred per patient. In all patients, the luteal phase was supported by Cyclogest (Actover, Alpharma, England) a vaginal progesterone at a dose of 400mg/Bid, which started from the day of oocyte retrieval. In cases where chemical pregnancy was detected two weeks following embryo transfer, clinical pregnancy was confirmed with ultrasound examination with the appearance of a gestational sac six weeks thereafter. 

Twin pregnancy rate was determined as the result of number of twins compared to total clinical pregnancies. Data regarding further course of pregnancies i.e miscarriage and live birth rates (number of live births per clinical pregnancy) were included in the study. 

**Primary outcome**

The primary outcome consisted of mean number of mature oocytes retrieved. 

**Secondary outcome**

Secondary outcomes included total number and top quality embryos and clinical pregnancy rate in PCOS patients.

**Statistical analysis**

Results were expressed as mean±standard deviation. Student’s t test was used to evaluate the differences between groups. Logistic regression model was used to assess the simultaneous effect of variables on ovary response. p<0.05 was considered statistically significant. Data were analyzed using SPSS software version 15.

## Results

**Figure 1:**
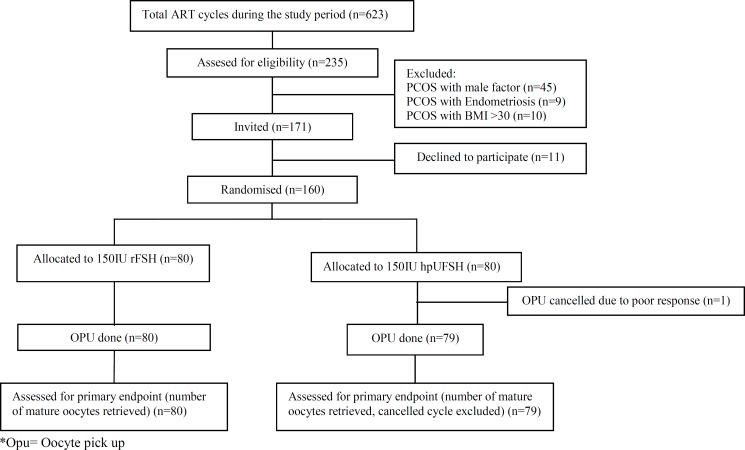
Consort flow chart of RCT.

The Consort flow chart concerning participant selection of the trial is shown in Figure 1. Both groups had similar demographic and basic characteristics including age, BMI, type and duration of infertility and baseline hormonal profiles (Table I). Considering the criteria of PCOS in the study groups before intervention, the two groups were matched as shown in table II. From the total of 160 PCOS patients studied, 159 cases resulted in embryo transfer. 

One patient in group B showed no response to ovulation induction and was therefore excluded from the study. Out of 159 patients, 69 (43.3%) achieved clinical pregnancy with 33 (41.2%) in group A and 36 cases (45%) in group B. The primary and secondary outcomes are shown in table III. There was no significant difference in the number of mature (metaphase II) oocytes, total number and top quality embryos, clinical pregnancy and live birth rates between the two treatment groups (Table III).

**Table I:** Demographic and basic characteristics of patients

**Variable**		**Group A**	**Group B**	**p-value**
Age* (mean ± SD)		31.29 ± 3.74	31.16 ± 2.65	0.80
BMI* (mean ± SD)		26.51±1.12	26.65±1.30	0.87
Infertility type**:				0.33
	- Primary [n (%)]	59 (73.8%)	58 (72.5%)	
	- Secondary [n (%)]	21 (26.2%)	22 (27.5%)	0.91
Infertility period* (years)		8.09 ± 3.31	8.51 ± 2.5	0.36
Hormonal profile:*				
	- FSH (IU/ml)	5.41 ± 1.98	5.30 ± 1.31	0.69
	- LH (IU/ml)	10.68 ± 3.99	10.26 ± 3.56	0.91
	- Testosterone (pg/dl)	1.06 ± 0.41	1.07 ± 0.40	0.95

* t- student test.

** chi-square.

**Table II:** Comparison of criteria of PCOS in the two study groups before intervention

**Variable**		**Group A** **n (%)**	**Group B** **n (%)**	**p-value**
Menstrual status				
	Regular	27 (33.75)	26 (32.5)	0.72
	Irregular	44 (55)	48 (60)	
	Oligomenorrhea	6 (7.5)	5 (6.2)	
	Amenorrhea	3 (3.75)	1 (1.3)	
Ovary size				
	Greater than 10 (ML)	17 (21.2)	15 (18.8)	0.69
	Smaller than 10 (ML)	63 (78.8)	65 (81.2)	
Number of follicles				
	≥ 10	62 (77.5)	61 (76.3)	0.85
	<10 follicles	18 (22.5)	19 (23.7)	
Hirsutism				
	Yes	37 (46)	37 (46)	0.92
	No	43 (54)	43 (54)	

Chi-square.

**Table III:** Comparison of variables in both groups after intervention

**Variable**		**Group A** **(mean ± SD)**	**Group B** **(mean ± SD)**	**p-value**
Number of gonadotropin ampoules used*** (mean ± SD)		20.35 ± 5.14	20.23 ± 3.85	0.87
Number of follicles 18 mm *** ( day of HCG)		3.72 ± 1.72	3.78 ± 1.55	0.81
Follicles 12 -14 mm***( day of HCG)		10.8 ± 3.36	10.97 ± 2.89	0.72
Endometrial thickness on hCG day (mm) ***		9.66 ± 1.67	10.36 ± 1.35	0.004
No. of retrieved oocytes***		13.03 ± 5.56	14.17 ± 4.89	0.17
No. of mature (M_II_) oocytes***		9.55±4.37	10.25±3.96	0.29
No. of total embryos ***		7.42 ± 3.35	7.63 ± 3.28	0.68
Fertilization rate (%)***		83.4±46.6	75.6±14.5	0.14
Embryo quality *				
	- A	63 (78.8%)	68 (85%)	0.26
	- B	14 (17.51%)	12 (15%)	
	- C or D	3 (3.8%)	0	
No. of top quality embryos per patient		3.59±.28	4.1±.24	0.12
No. of embryos transferred ***		2.05 ± 0.72	1.97 ± 0.31	0.40
No of embryos cryo preserved ***		310	325	0.9
No. of frozen embryos per patient***		3.91±2.66	4.06±2.56	
Chemical pregnancy *(positive HCG)		36 (45%)	37 (46.25%)	0.8
Clinical pregnancy* (gestational sac)		33 (41.2%)	36 (45%)	0.67
Ongoing Pregnancy* (more than 12 weeks)		24 (30%)	29 (36.3%)	0.67
Miscarriage rate *		9 (11.2%)	7 (8.7%)	0.67
OHSS **				
	-Slight	4 (5%)	5 (6.25%)	0.9
	- Moderate to severe	2 (2.5%)	2 (2.5%)	
Twin pregnancy rate **		4 (12.12%)	6 (16.66%)	0.81
Live birth rate **		17 (21.25%)	19 (23.75%)	0.8

* Fisher Exact test.

** Chi-square.

*** T-test.

*Opu= Oocyte pick up

## Discussion

The aim of the present study was to compare recombinant FSH with highly purified urinary FSH in PCOS patients who usually have higher levels of LH and therefore FSH alone regimens are mostly preferred (17). The development of various preparations of gonadotropins plays an important role in the treatment of human infertility and has provided clinicians with the possibility to choose the most appropriate regimens individually tailored to patient conditions. 

Different clinical trials and meta-analyses have been performed in order to distinguish the significant advantages of the present products including human menopausal gonadotropin (hMG), uFSH-HP and recombinant FSH in ART cycles with different results (18-26). Most of these studies have been done on non PCOS patients and there is apparently no specific evidence concerning the most appropriate gonadotropins to be used for controlled ovarian hyperstimulation in PCOS.

The results of our study showed that highly purified urinary and recombinant FSH have similar clinical efficacy regarding the mean number of oocytes, grade A embryos transferred and clinical pregnancy rate in PCOS patients. The total clinical pregnancy rate in this study (43.4%) is higher than the usual ART outcome ( 35%) in our center. These findings are close to the results of a review performed by Al-Inany *et al* (21). In their meta-analysis on 20 randomized clinical trials (46,170 IVF cycles) in which PCOS patients were usually excluded, comparing urinary FSH and recombinant FSH they showed similar clinical and ongoing pregnancy rates (more than 12 weeks gestation).

In a clinical trial by Abate *et al* comparing human follicle stimulating hormone (hFSH) and recombinant FSH (rFSH) on 401 women in ART, no significant difference in oocyte/embryo quality was observed between the two groups. The number of oocytes retrieved was significantly higher in the hFSH group. Fertilization, cleavage and implantation rates, pregnancy and miscarriage rates were similar in both groups. This study demonstrated that hFSH and rFSH products are equivalent in terms of clinical efficacy (22). In another study by Selman *et al* on 267 patients in IVF-ICSI cycles, pregnancy and implantation rates were non-significantly higher in the urinary FSH compared to the recombinant FSH group. The grade 1 embryo score was significantly higher in the urinary FSH than the recombinant FSH group, and the live birth rate was non-significantly higher in the former group. They concluded that purified urinary FSH is as effective, efficient, and safe for clinical use as recombinant FSH (23). 

Different results have been achieved in a meta-analysis by Manassiev who examined in five randomized clinical trial, the effectiveness of r-FSH compared to u-FSH in increasing pregnancy rate in a total of 65 patients treated with r-FSH and 627 treated with u-FSH. When all studies were combined and analyzed together, the use of recombinant FSH led to significant improvement in clinical pregnancy rate. They concluded that recombinant FSH appears to be more effective than urinary FSH in achieving clinical pregnancy in IVF-ET cycles. However, the results should be interpreted with caution because of the small size of the individual studies (24). 

In a study by Balen *et al* highly purified urinary FSH was compared with recombinant FSH to evaluate induction ovulation results using a low-dose step-up protocol in 151 PCOS patients who were resistant to clomiphene citrate. The ovulation rate was 85.2% with HP-FSH and 90.9% with rFSH. No differences were noted between groups in number of follicles ≥12mm, ≥15mm or ≥18mm, mono-follicular development, pregnancy rates, endometrial thickness, number of ovarian stimulation syndrome cases (25). 

In a meta-analysis performed by Bayram *et al,* in order to compare the safety, effectiveness in terms of ovulation, pregnancy, miscarriage, multiple pregnancy rate and ovarian hyper-stimulation syndrome (OHSS) in women with clomiphene-resistant polycystic ovary syndrome (PCOS) who had used recombinant FSH or urinary FSH, four randomized trials were identified. No significant differences were demonstrated for the relevant outcomes. (26).

In the present study, the endometrial thickness in group B who received highly purified urinary FSH was significantly higher than the other group. (10.36±1.35 vs. 9.66±1.67 mm, p=0.004)

Despite the statistical difference, the average endometrial thickness in both was in a normal range and therefore, it did not affect the pregnancy rate (clinical and chemical).

In accordance with this finding in a retrospective study performed by Corbacioğlu, the pregnancy rates were compared in 241 ART cycles. The cycles were classified into three groups according to ultra-sonographic endometrial thickness measurements on the day of hCG application with 51 cases (group 1) ≤8mm, 182 cases (group 2) between 8-14 mm, and 8 cases (group 3) 14 mm. There was no significant difference in pregnancy rates between the three endometrial thickness groups. They concluded that endometrial thickness is not a useful parameter in predicting implantation and conception rates in ART cycles (27).

Different results in various studies are perhaps due to biological differences in patients, dosage of drugs consumed and study designs. Pharmaco-dynamic and pharmacokinetic studies have also confirmed that a broad diversity exists among individuals in response to urinary and recombinant FSH primarily because of individual ovarian sensitivity to FSH (28).

## Conclusion

Significant difference in the average number of follicles, oocytes, embryos transferred, grade A embryos, chemical and clinical pregnancy in the two groups of highly purified urinary and recombinant FSH treatment was not shown in this study. Considering the results, it seems that both FSH alone products can be used for controlled ovarian hyper stimulation in patients with PCOS with similar ART outcomes. Therefore either compound can be used according to availability and patient acceptance. 

## Footnote

***Registration ID in IRCT: IRCT138901193636NI***
